# Association between light exposure and sleep problems related to nocturia in older adults: the Nagahama study

**DOI:** 10.1186/s40101-026-00429-7

**Published:** 2026-04-08

**Authors:** Isuzu Nakamoto, Sayaka Uiji, Hiromitsu Negoro, Takayuki Yoshino, Sachiko Horita, Kazuya Setoh, Yasuharu Tabara, Kimihiko Murase, Takuma Minami, Takeshi Matsumoto, Yoshinari Nakatsuka, Satoshi Hamada, Naomi Takahashi, Naoko Komenami, Fumihiko Matsuda, Kazuo Chin, Tomoko Wakamura, The Nagahama Study group

**Affiliations:** 1 Human Health Sciences, Graduate School of Medicine, Kyoto University, Kyoto, Japan; 2 The Tokyo Metropolitan Support Center for Preventative Long-Term and Frail Elderly Care, Tokyo Metropolitan Institute for Geriatrics and Gerontology, Tokyo, Japan; 3 Department of Urology, Institute of Medicine, University of Tsukuba, Ibaraki, Japan; 4 Department of Epidemiology for Community Health and Medicine, Kyoto Prefectural University of Medicine, Kyoto, Japan; 5 Graduate School of Public Health, Shizuoka Graduate University of Public Health, Shizuoka, Japan; 6 Center for Genomic Medicine, Kyoto University Graduate School of Medicine, Kyoto, Japan; 7 Department of Advanced Medicine for Respiratory Failure, Kyoto University Graduate School of Medicine, Kyoto, Japan; 8 Department of Respiratory Medicine, Kyoto University Graduate School of Medicine, Kyoto, Japan; 9 Department of Primary Care and Emergency Medicine, Graduate School of Medicine, Kyoto University, Kyoto, Japan; 10 Department of Respiratory Medicine, Saiseikai-Noe Hospital, Osaka, Japan; 11 Department of Food and Nutrition, Kyoto Women’s University, Kyoto, Japan; 12 Department of Sleep Medicine and Respiratory Care, Division of Respiratory Medicine, Department of Internal Medicine, Nihon University, Tokyo, Japan

**Keywords:** Nocturnal voiding frequency, Light exposure, Persons aged 65 years and older

## Abstract

**Background:**

The prevalence of nocturia-related sleep problems is higher in older people. This study investigated whether morning and evening light exposure is associated with the sleep problems related to objectively identified nocturnal urination in community-dwelling older adults.

**Methods:**

This cross-sectional study analyzed data collected between 2013 and 2016 for the Nagahama Prospective Cohort for Comprehensive Human Bioscience (the Nagahama Study). The study included 1585 participants aged ≥ 65 years who met the criteria for inclusion in the present analysis. An actigraph was used to measure sleep, nocturnal voiding time, and light exposure. The frequency of nocturnal voiding was derived from self-reported entries in the participants’ sleep diaries. Morning light (ML) exposure was defined as the period between waking and 12:00, while evening light (EL) exposure was defined as the period between 18:00 and bedtime. The first uninterrupted sleep period (FUSP) was calculated as the interval between the sleep onset time and the first nocturnal void. Additionally, the ratio of FUSP to sleep period time (FUSP/SPT) was calculated. Associations between light exposure parameters and the frequency of nocturnal voiding, FUSP, or FUSP/SPT were examined using multivariable linear regression models.

**Results:**

Of the 1585 participants, 38.7% were male median age was 70 years. The median nocturnal voiding frequency was 1.0 times (IQR: 0.4, 1.4) and the median FUSP was 4.6 h (IQR: 3.2, 5.9). In multivariable linear regression, log ML exposure was significantly associated with FUSP and FUSP/SPT (B = 0.17, 95% CI: 0.02, 0.32; B = 2.20, 95% CI: 0.26, 4.14, respectively). In contrast, the duration of ML exposure ≥ 1,000 lx, EL exposure, and the duration of EL exposure ≥ 50 lx were not significantly associated with the frequency of nocturnal voiding, FUSP or FUSP/SPT.

**Conclusions:**

Greater morning light exposure was associated with a longer FUSP. Morning bright light exposure may therefore help delay nocturia-related sleep interruptions, potentially contributing to improved sleep quality among older people.

**Supplementary Information:**

The online version contains supplementary material available at 10.1186/s40101-026-00429-7.

## Background

The prevalence of nocturia occurring ≥ 1 time/night is 69–77% [1, 2] and increases with age [2]. Nocturia disrupts sleep, often hindering falling back asleep and potentially causing insomnia. Its frequency [3] and the duration of uninterrupted sleep, specifically the first uninterrupted sleep period (FUSP) [4], are therefore paid particular attention. It has been reported that the longer the duration until the first nocturnal urination (indicating longer continuous sleep), the higher the sleep quality [5, 6].

Various lifestyle improvement methods are implemented to improve nocturia, such as not drinking large amounts of water and refraining from caffeine or alcohol intake before bed, reducing salt intake, and receiving before-bedtime passive body heating [7–9]. Treating nocturia, however, remains challenging, even with pharmacotherapy or surgical interventions for benign prostatic hyperplasia [10]. While lifestyle modifications play an essential role in improving nocturia, they can impose a burden on patients and lead to compliance issues. Consequently, less burdensome approaches are needed. It is increasingly recognized that urination follows a circadian rhythm, the disruption of which is associated with nocturia [11, 12]. Exposure to daytime bright light advances the peak timing of the circadian rhythm of urine production, promoting increased excretion during daytime [13]. Therefore, we hypothesized that morning bright light exposure may contribute to improving sleep problems related to nocturnal urination. This study investigated whether morning and evening light exposure is associated with sleep problems related to nocturnal urination in older adults.

## Methods

### Study design and participants

**Fig. 1:**
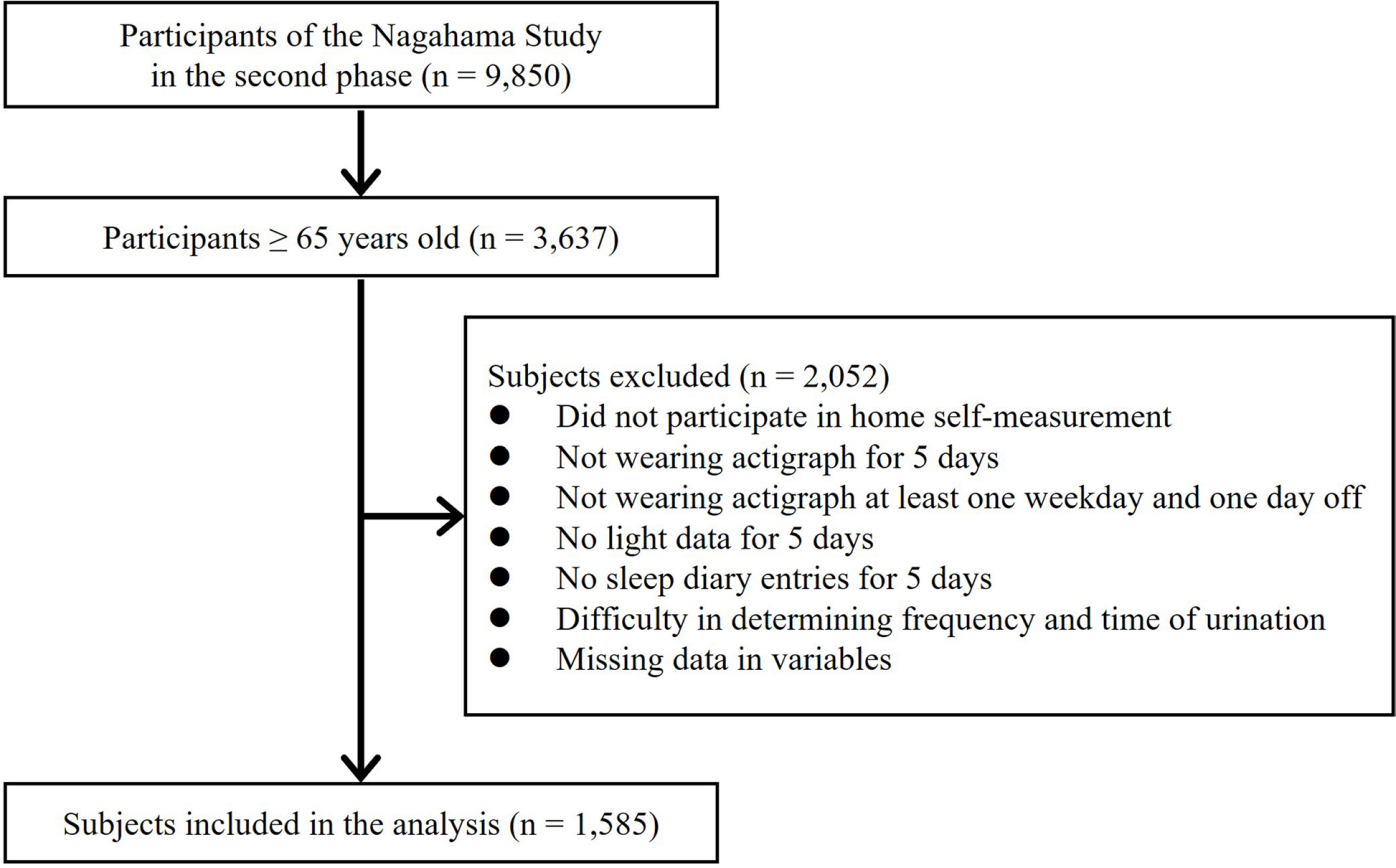
Flowchart of the selection of study participants from the Nagahama Study

This cross-sectional study was conducted using survey data from the Nagahama Prospective Cohort for Comprehensive Human Bioscience (the Nagahama Study), collected between 2013 and 2016 [14]. Participants in this study were recruited from the general population of Nagahama, a city of 125,000 inhabitants in Shiga Prefecture, Japan. The second phase of the Nagahama Study recruited 9,850 community residents between 30 and 74 years old who did not have serious physical disabilities or health problems. The participants included in the present study were aged 65 years and older. Figure 1 presents the details of the sample selected for this study’s analysis. The ethics committee of Kyoto University Graduate School of Medicine (No. G278) and the Nagahama Municipal Review Board approved the study protocol. All participants provided written informed consent.

### Self-measurements at home

Once each participant had visited the examination site, a 1-week self-measurement period commenced. The participants were instructed to wear an actigraph on their non-dominant wrist for the week and a pulse oximeter for the first four nights after the site visit. They were also asked to complete a sleep diary during the same period.

### Exposure variables: measurement of light exposure

An actigraph (Actiwatch 2 or Actiwatch Spectrum Plus; Philips Respironics, Inc., Murrysville, Pennsylvania, USA) was used to measure light exposure in 1-min intervals over 7 days. This device simultaneously measures physical activity and illuminance using a built-in three-dimensional accelerometer and a silicon photodiode, respectively. It includes a photodiode with a spectral sensitivity similar to that of the human eye (luminance sensitivity, 0–150,000 lx). The participants were instructed to pull the silicon photodiode part of the device out of their shirtsleeves as much as possible. Daytime light intensity readings < 1 lx were considered artifact data because of sensor coverage and were excluded from the calculations [15]. If more than half of the daytime light data were interpreted as artifacts, they were treated as missing data in accordance with previous research [15].

Morning light (ML) exposure was defined as the period between waking and 12:00, while evening light (EL) exposure was defined as the period between 18:00 and bedtime. If the average timing of nighttime melatonin secretion is approximately 04:00, light exposure after 18:00 will delay the phase, and light exposure after 06:00 will advance the phase [16]; therefore, this study focused on light exposure in the morning and evening. We calculated the average light exposure (illuminance) during the morning and evening periods.

Previous studies have reported that the degree of melatonin suppression varies depending on light intensity. This study therefore set the threshold for ML exposure at 1,000 lx or more and for EL exposure at 50 lx or more [17], measuring durations of ML exposure ≥ 1,000 lx and EL exposure ≥ 50 lx.

### Outcome: sleep problems related to objectively identified nocturnal urination

Dedicated software (Actiware version 6.0.1; Philips Respironics) was used to automatically determine asleep or awake states from actigraph data based on the underlying physical activity during ± 2 min intervals. Trained investigators calculated bed-in and bed-out times and sleep duration from the actigraph and by referring to the sleep diaries, if available, and the illuminance data. The means across all days were used for the analysis. Data for ≥ 5 nights (including at least 1 weekend night) were required for inclusion in the analysis, and the values were averaged. Participants recorded the nocturnal voiding frequency in their sleep diaries for 7 days.

Instances of increased actigraph activity and light exposure were taken as the timing of nocturnal urination. If the frequency of increased activity and light exposure did not correspond to the sleep diary entries, the timing of nocturnal urination was considered indeterminate. FUSP was calculated as the interval between the timing of the first nocturnal void and the sleep onset time. For nights without nocturnal urination, FUSP was defined as the sleep period time (SPT) for that night. Because the length of the FUSP is affected by SPT, the ratio of FUSP to SPT (FUSP/SPT) was calculated.

### Covariate variables

Urologists, sleep specialists, and chronobiologists discussed the nocturia-related variables—sleep and light exposure—and identified the following as covariate variables.

#### Lifestyle-related parameters

Information on smoking, drinking, and exercise habits was collected using a structured self-administered questionnaire. Drinking habits were assessed based on the frequency per week. Exercise habits were assessed using a questionnaire item asking, "Do you walk or engage in similar physical activity for at least one hour a day?" Respondents were given two response options: "Yes" and "No."

#### Sleep-related parameters

As objective sleep parameters, sleep onset time (SOT) and wake time (WT) were measured using actigraphy. We obtained the actigraphy-modified 3% oxygen desaturation index (Acti-ODI3%) from the 3% oxygen desaturation index and actual sleep duration using the actigraph. Oxygen saturation (SpO2) was measured using pulse oximetry (PULSOXMe300; Konica Minolta, Inc., Tokyo, Japan). Acti-ODI3% was used as an indicator of sleep apnea syndrome. Sleeping pill use data were collected using a structured self-administered questionnaire. Subjective sleep quality was assessed using the Pittsburgh Sleep Quality Index (PSQI), with higher scores indicating poorer sleep quality (range: 0–21) [18, 19]. A score of 6 or higher was used to define the presence of sleep disorders.

#### Urology-related parameters

Nocturia-associated symptoms were evaluated using the Overactive Bladder Symptom Score (OABSS), with higher scores indicating greater symptom severity (range: 0–15) [20]. Scores were categorized into three levels of severity: mild (≤ 5), moderate (6–11), and severe (≥ 12). Additionally, lower urinary tract symptoms (LUTS) were assessed using the International Prostate Symptom Score (IPSS), with higher scores reflecting more severe symptoms (range: 0–35) [21, 22]. Scores were categorized into three levels of severity: mild (≤ 7), moderate (8–19), and severe (≥ 20). The estimated glomerular filtration rate (eGFR) served as an indicator of kidney function and was calculated using the equation proposed by the Japanese Society of Nephrology: estimated glomerular filtration rate = 194 × serum creatinine^−1.094^ × age^−0.287^ (× 0.739 for women) [23]. In addition, levels of a hormone related to nocturia, plasma B-type natriuretic peptide (BNP), were measured [24]. BNP and eGFR were analyzed from blood samples collected on the day of the survey.

#### Other diseases and health-related parameters

Anthropometric measurements (height and weight) and resting blood pressure were recorded on the day of the survey. Body weight and height were used to compute the body mass index (BMI). Participants were considered to have hypertension if they had systolic blood pressure (SBP) > 140 mmHg, diastolic blood pressure (DBP) > 90 mmHg, or were taking antihypertensive medication. Diabetes was determined as having glycated hemoglobin (HbA1c) > 6.5% or undergoing treatment with oral antihyperglycemic agents and/or insulin. HbA1c was analyzed using blood samples collected on the day of the survey. Self-rated health was assessed using the question, "How do you feel about your health status?" with four response options—"very good," "good," "fair," and "poor"—which were categorized into two groups for data analysis—"good" (combining "very good" and "good") and "poor" (combining "fair" and "poor").

#### Other variables

Demographic information (sex, age, living arrangement, educational attainment, and household income) was collected using a self-administered questionnaire. The daylength (sunrise to sunset) was determined based on the first day of measurements in Nagahama city from the National Astronomical Observatory of Japan website (https://eco.mtk.nao.ac.jp/cgi-bin/koyomi/koyomiy.cgi).

### Statistical analysis

Data for continuous variables are presented as medians with interquartile ranges (IQRs), and categorical variables are presented as frequencies and percentages. Multiple linear regression analyses were performed using the nocturnal voiding frequency, FUSP, and FUSP/SPT as outcome variables and the light exposure variables as exposure variables, adjusting for all covariates. Non-normally distributed continuous variables, as identified by histograms, were log-transformed or categorized for use in multivariate analysis. Average light exposure (ML exposure and EL exposure), the durations of ML exposure ≥ 1,000 lx and EL exposure ≥ 50 lx, Acti-ODI3%, and BNP were log-transformed due to their non-normal distribution. Statistical analyses were conducted using IBM SPSS Statistics for Windows, Version 30.0 (IBM Corp., Armonk, NY, USA). A *P*-value < 0.05 was considered statistically significant.

## Results

Of the 1,585 participants, 38.7% were male and median age was 70 years (IQR: 67, 73) (Table 1). The median nocturnal voiding frequency was 1.0 times (IQR: 0.4, 1.4). The median FUSP was 4.6 h (IQR: 3.2, 5.9) and FUSP/SPT was 66.5% (IQR: 44.5, 86.9). Regarding light exposure variables, the median ML exposure was 681 lx (IQR: 385, 1210) and the median EL exposure was 27 lx (IQR: 18, 40). The median durations of ML exposure ≥ 1,000 lx was 45.5 min (IQR: 25.3, 72.3), while the duration of EL exposure ≥ 50 lx was 32.2 min (IQR: 18.2, 50.3).

**Table 1:** Characteristics of the study participants

	All (*n* = 1585)	Male (*n* = 613)	Female (*n* = 972)
Age (y), median (IQR)	70 (67, 73)	71 (68, 74)	70 (67, 73)
Living arrangement, Living with cohabitants, n (%)	1496 (94.4)	599 (97.7)	897 (92.3)
Educational attainment (y), median (IQR)	12 (9, 12)	12 (9, 13)	12 (9, 12)
Household income, n (%)			
< 2 million yen	338 (21.3)	94 (15.3)	244 (25.1)
2–4 million yen	791 (49.9)	342 (55.8)	449 (46.2)
4–6 million yen	266 (16.8)	108 (17.6)	158 (16.3)
6–8 million yen	97 (6.1)	33 (5.4)	64 (6.6)
≥ 8 million yen	93 (5.9)	36 (5.9)	57 (5.9)
Daylight hours (hours), median (IQR)	5.2 (3.1, 6.3)	5.4 (3.4, 6.6)	4.6 (3.1, 6.3)
Current smoker, Smoking, n (%)	86 (5.4)	75 (12.2)	11 (1.1)
Drinking frequency (days/week), median (IQR)	0 (0, 4)	4 (0, 7)	0 (0, 0)
Physical activity, Regular exercise, n (%)	993 (62.6)	374 (61)	619 (63.7)
BMI, median (IQR)	22.3 (20.4, 24.2)	22.7 (21.1, 24.4)	21.9 (20.1, 24)
Subjective health status, Good health, n (%)	1310 (82.6)	504 (82.2)	806 (82.9)
Diabetes mellitus, n (%)	179 (11.3)	95 (15.5)	84 (8.6)
Hypertension, n (%)	934 (58.9)	405 (66.1)	529 (54.4)
Sleep medication use, n (%)	193 (12.2)	53 (8.6)	140 (14.4)
Sleep onset time (clock time), median (IQR)	22.8 (22.1, 23.4)	22.6 (22, 23.3)	22.8 (22.2, 23.5)
Wake time (clock time), median (IQR)	5.9 (5.3, 6.4)	5.8 (5.2, 6.4)	5.9 (5.4, 6.4)
Acti-ODI3% (events/h), median (IQR)	8.4 (5.3, 13.1)	11 (6.7, 16.7)	7.3 (4.8, 11.2)
PSQI, median (IQR)	5 (3, 7)	4 (3, 6)	5 (3, 7)
No sleep disorder (≤ 5), n (%)	987 (62.3)	415 (67.7)	572 (58.8)
Sleep disorder (≥ 6), n (%)	598 (37.7)	198 (32.3)	400 (41.2)
eGFR (mL/min/1.73m^2^), median (IQR)	70.2 (62.6, 78.9)	68.7 (61.1, 76.5)	71.3 (63.8, 80)
BNP, median (IQR)	21.5 (13.7, 34.5)	21.6 (13, 38.2)	21.4 (14.1, 33.3)
IPSS, median (IQR)	4 (2, 8)	6 (3, 13)	3 (1, 6)
Mild (≤ 7), n (%)	1143 (72.1)	346 (56.4)	797 (82.0)
Moderate (8–19), n (%)	358 (22.6)	207 (33.8)	151 (15.5)
Severe (≥ 20), n (%)	84 (5.3)	60 (9.8)	24 (2.5)
OABSS, median (IQR)	2 (1, 4)	2 (1, 4)	2 (1, 4)
Mild (≤ 5), n (%)	1399 (88.3)	538 (87.8)	861 (88.6)
Moderate (6–11), n (%)	180 (11.4)	72 (11.7)	108 (11.1)
Severe (≥ 12), n (%)	6 (0.4)	3 (0.5)	3 (0.3)
Nocturnal voiding frequency (times/day), median (IQR)	1.0 (0.4, 1.4)	1.0 (0.6, 1.7)	0.9 (0.3, 1.3)
FUSP (h), median (IQR)	4.6 (3.2, 5.9)	4.2 (2.9, 5.5)	4.7 (3.4, 6)
FUSP/SPT (%), median (IQR)	66.5 (44.5, 86.9)	61.2 (40.6, 81.5)	70.4 (47.5, 89.7)
ML exposure (lx), median (IQR)	681 (385, 1210)	1102 (637, 1700)	537 (313, 866)
EL exposure (lx), median (IQR)	27 (18, 40)	32 (22, 52)	23 (16, 35)
the duration of ML exposure ≥ 1,000 lx (m), median (IQR)	45.5 (25.3, 72.3)	63.8 (39.2, 95)	37.1 (20.8, 55.8)
the duration of EL exposure ≥ 50 lx (m), median (IQR)	32.2 (18.2, 50.3)	39.5 (22.6, 59.6)	28.3 (15.8, 43)

Data presented as n (%) or median (first quartile, third quartile)

*BMI* body mass index, *PSQI* the Pittsburgh Sleep Quality Index, *Acti-ODI3%* the actigraphy-modified 3% oxygen desaturation index, *eGFR* estimated glomerular filtration rate, *BNP* B-type natriuretic peptide, *IPSS* the International Prostate Symptom Score, *OABSS* the Overactive Bladder Symptom Score, *FUSP* the first uninterrupted sleep period, *SPT* sleep period time, *ML* morning light, *EL* evening light

Table 2 presents the associations between light exposure parameters and sleep problems related to nocturnal urination. Log ML exposure was significantly associated with FUSP and FUSP/SPT (B = 0.17, 95% CI: 0.02, 0.32; B = 2.20, 95% CI: 0.26, 4.14, respectively). In contrast, log the duration of ML exposure ≥ 1,000 lx was not significantly associated with FUSP and FUSP/SPT. For Evening light, neither EL exposure nor the duration of EL exposure ≥ 50 lx showed significant associations with FUSP. None of the light exposure parameters (ML or EL, either total exposure or duration above the thresholds) were associated with nocturnal voiding frequency. Supplementary tables 1–4 contain the detailed results of the multivariable analyses.

**Table 2:** Association between sleep problems related to nocturnal urination and light exposure parameters

	B (95%CI)	P
Outcome: Nocturnal voiding frequency		
Log ML exposure^a^	0.02 (−0.09, 0.13)	0.76
Log the duration of ML exposure ≥ 1,000 lx^a^	0.02 (−0.09, 0.14)	0.69
Log EL exposure^a^	−0.06 (−0.18, 0.06)	0.33
Log the duration of EL exposure ≥ 50 lx^a^	−0.04 (−0.14, 0.05)	0.37
Outcome: FUSP		
Log ML exposure ^b^	0.17 (0.02, 0.32)	0.02
Log the duration of ML exposure ≥ 1,000 lx ^b^	0.05 (−0.11, 0.21)	0.54
Log EL exposure ^b^	0.02 (−0.15, 0.18)	0.82
Log the duration of EL exposure ≥ 50 lx ^b^	0.08 (−0.05, 0.21)	0.22
Outcome: FUSP/SPT		
Log ML exposure ^b^	2.20 (0.26, 4.14)	0.03
Log the duration of ML exposure ≥ 1,000 lx ^b^	0.56 (−1.50, 2.62)	0.59
Log EL exposure ^b^	0.02 (−2.13, 2.18)	0.98
Log the duration of EL exposure ≥ 50 lx ^b^	0.60 (−1.09, 2.28)	0.49

^a^ Adjusted for age, sex, living arrangement, educational attainment, household income, daylight hours, current smoker, drinking frequency, physical activity, BMI, subjective health status, diabetes mellitus, hypertension, sleep medication use, sleep onset time, wake time, the actigraphy-modified 3% oxygen desaturation index (Acti-ODI3%), the Pittsburgh Sleep Quality Index (PSQI), estimated glomerular filtration rate (eGFR), B-type natriuretic peptide (BNP), the International Prostate Symptom Score (IPSS), and the Overactive Bladder Symptom Score (OABSS)

^b^a + nocturnal voiding frequency

*FUSP* the first uninterrupted sleep period, *SPT* sleep period time, *ML* morning light, *EL* evening light

## Discussion

In this study, morning light exposure was associated with a longer FUSP and a higher FUSP/SPT. These findings suggest that morning bright light exposure may play a role in promoting consolidated sleep in older people.

Given the high prevalence of nocturia in the older population, prolonging the FUSP represents an essential clinical objective for improving sleep quality. A previous study reported that a shorter FUSP was associated with lower sleep quality [5]. Regarding clinical treatment, desmopressin significantly prolonged the FUSP in patients with nocturia caused by nocturnal polyuria [25]. However, only a limited number of patients benefitted from desmopressin, and concerns remain regarding its potential side effects [26]. The findings of this study are highly relevant, as morning light exposure represents a practical and low-burden strategy for integration into the daily routines of older adults. Exposure to high-intensity light in the morning advances the phase of the circadian rhythm [16] and improves sleep quality [27, 28]. It has also been reported that high-intensity light exposure during the day can influence the rhythm of urine production [13]. Together, this research suggests that exposure to light in the morning is associated with both sleep and the circadian rhythm of urine production, potentially long FUSP.

Log ML exposure, but not log duration of ML exposure ≥ 1,000 lx, was significantly associated with FUSP and FUSP/SPT. The total amount of morning light exposure may reflect the actual circadian stimulus more accurately than the duration spent above a specific high-intensity threshold, thereby showing a stronger association with circadian entrainment and sleep maintenance. In contrast, a prior study of community-dwelling older adults reported that a longer duration of light exposure ≥ 1,000 lx during the period from waking to bedtime was significantly associated with increased urinary 6-sulfatoxymelatonin excretion [29] and shorter wake after sleep onset [15]. However, that study defined daytime light exposure as the entire out-of-bed period, whereas the present study assessed light exposure during the morning (from waking to 12:00). While few studies have reported daytime light levels in older adults, a prior study indicated exposure to high-intensity light (≥ 1,000 lx) between 12:00 and 18:00 [30]. Future research is required to examine the associations with light exposure during this 12:00–18:00 period, which was not addressed in the present study.

In this study, no association was observed between evening light exposure and FUSP. Evening light exposure delays circadian rhythms [16]. Nighttime light exposure reduces melatonin production and decreases sleep quality in older people [31]. Furthermore, nighttime melatonin excretion has been reported to be inversely correlated with nocturia [29]. Previous studies on light exposure among community-dwelling older adults have defined daytime light exposure as the period from waking to bedtime and have examined its associations with various outcomes [15]. Further research is needed to clarify the specific evening light exposure periods that influence circadian rhythms in older adults.

Morning light exposure was not associated with nocturnal voiding frequency in this study. However, this does not preclude an association with physiological factors related to the timing of the first nocturnal void, such as initial functional bladder capacity, produced urine volume, or arousal threshold, which may be better reflected by the FUSP. Further studies are required to elucidate the mechanisms linking morning light exposure to FUSP.

This study has several limitations. First, urination timing was estimated based on changes in activity level and illuminance, which may not accurately reflect the actual time of urination, nocturnal urine volume, or nocturnal single-voided volume. Second, as a cross-sectional study, it was not possible to establish a causal relationship between light exposure and FUSP. Third, illuminance was measured using an activity meter worn on the arm, which may not correspond to the illuminance levels near the eyes. Fourth, the time windows for light exposure in this study, which account for effects on circadian rhythms, were established based on research findings in younger populations [16] due to the lack of prior studies targeting older adults. Therefore, the validity of these time settings for the older people has not yet been verified. Additionally, as a general limitation of observational studies, the potential influence of other unknown confounders that may be strongly associated with the observed relationships remains unclear.

## Conclusions

This study suggests that greater morning light exposure is associated with a longer first uninterrupted sleep period (FUSP) and a higher FUSP/SPT among older people. The results of this study suggest that morning light exposure may be associated with the improvement of sleep problems related to nocturnal voiding.

## Supplementary Information

Supplementary Material 1.

Supplementary Material 2.

Supplementary Material 3.

Supplementary Material 4.

Supplementary Material 5.

## Data Availability

No datasets were generated or analysed during the current study.

## Abbreviations

FUSP: First uninterrupted sleep period
SPT: Sleep period time
PSQI: Pittsburgh Sleep Quality Index
Acti-ODI3%: Actigraphy-modified 3% oxygen desaturation index
eGFR: Estimated glomerular filtration rate
BNP: B-type natriuretic peptide
IPSS: International Prostate Symptom Score
OABSS: Overactive Bladder Symptom Score
ML: Morning light
EL: Evening light
