# Supplementary material for: Association between light exposure and sleep problems related to nocturia in older adults: the Nagahama study
Source: J Physiol Anthropol. 2026 Apr 8;45:11. doi: 10.1186/s40101-026-00429-7 (PMC13182062; doi:10.1186/s40101-026-00429-7)
Supplement: Supplementary file 1 — Supplementary Material 1. [file 40101_2026_429_MOESM1_ESM.docx]

| Supplementary table1. Association between sleep problems related to nocturnal urination and ML exposure. | | | | | | |
| --- | --- | --- | --- | --- | --- | --- |
|  | Nocturnal voiding frequency | | FUSP | | FUSP/SPT | |
|  | B (95%CI) | P | B (95%CI) | P | B (95%CI) | P |
| sex (Ref: Male) | -0.12 (-0.22, -0.03) | 0.01 | 0.04 (-0.10, 0.17) | 0.59 | 0.39 (-1.35, 2.14) | 0.66 |
| Age (y) | 0.02 (0.01, 0.03) | < 0.01 | 0.01 (-0.01, 0.02) | 0.34 | 0.09 (-0.07, 0.25) | 0.28 |
| Living arrangement, Living with cohabitants (Ref: Living alone) | 0.03 (-0.13, 0.18) | 0.74 | 0.04 (-0.17, 0.25) | 0.70 | 0.41 (-2.33, 3.16) | 0.77 |
| Educational attainment (y) | -0.01 (-0.03, 0.00) | 0.09 | 0.00 (-0.02, 0.02) | 0.96 | -0.01 (-0.30, 0.27) | 0.92 |
| Household income |  |  |  |  |  |  |
| < 2 million yen | Ref. |  |  |  |  |  |
| 2–4 million yen | 0.00 (-0.09, 0.1) | 0.93 | -0.01 (-0.13, 0.12) | 0.91 | -0.34 (-1.98, 1.3) | 0.68 |
| 4–6 million yen | -0.02 (-0.14, 0.09) | 0.68 | 0.07 (-0.09, 0.23) | 0.40 | 0.23 (-1.87, 2.33) | 0.83 |
| 6–8 million yen | -0.04 (-0.2, 0.12) | 0.59 | 0.09 (-0.13, 0.31) | 0.43 | 0.76 (-2.11, 3.63) | 0.60 |
| ≥ 8 million yen | 0.10 (-0.06, 0.26) | 0.23 | 0.29 (0.06, 0.51) | 0.01 | 2.86 (-0.05, 5.78) | 0.05 |
| Daylight hours (IQR) | 0.00 (-0.03, 0.03) | 0.92 | 0.04 (-0.01, 0.08) | 0.09 | 0.52 (-0.04, 1.08) | 0.07 |
| Current smoker, Smoking  (Ref: Not Smoking) | -0.17 (-0.32, -0.01) | 0.03 | 0.07 (-0.15, 0.28) | 0.53 | 0.64 (-2.16, 3.44) | 0.66 |
| Drinking frequency (days/week) | 0.00 (-0.02, 0.01) | 0.52 | 0.01 (-0.01, 0.03) | 0.19 | 0.14 (-0.13, 0.41) | 0.29 |
| Physical activity, Regular exercise (Ref: Not regular exercise) | 0.02 (-0.05, 0.09) | 0.58 | -0.01 (-0.11, 0.09) | 0.78 | -0.37 (-1.67, 0.93) | 0.58 |
| BMI | 0.00 (-0.01, 0.01) | 0.83 | 0.00 (-0.01, 0.02) | 0.68 | 0.07 (-0.16, 0.30) | 0.54 |
| Subjective health status,  Good health (Ref: Not health) | 0.08 (-0.02, 0.17) | 0.11 | -0.04 (-0.17, 0.09) | 0.56 | -0.60 (-2.29, 1.09) | 0.49 |
| Diabetes mellitus (Ref: Not diabetes mellitus) | 0.04 (-0.07, 0.15) | 0.43 | -0.08 (-0.23, 0.07) | 0.29 | -1.44 (-3.41, 0.53) | 0.15 |
| Hypertension (Ref: Not hypertension) | 0.03 (-0.04, 0.11) | 0.39 | 0.07 (-0.03, 0.17) | 0.19 | 0.95 (-0.37, 2.26) | 0.16 |
| Sleep medication use (Ref: Not sleep medication use) | -0.02 (-0.14, 0.10) | 0.73 | -0.19 (-0.35, -0.03) | 0.02 | -2.30 (-4.36, -0.23) | 0.03 |
| Sleep onset time (clock time) | -0.24 (-0.28, -0.20) | < 0.01 | -0.54 (-0.6, -0.48) | < 0.01 | 1.65 (0.87, 2.43) | < 0.01 |
| Wake time (clock time) | 0.13 (0.08, 0.17) | < 0.01 | 0.43 (0.37, 0.49) | < 0.01 | -2.96 (-3.78, -2.15) | < 0.01 |
| Log Acti-ODI3% | 0.31 (0.18, 0.45) | < 0.01 | -0.08 (-0.26, 0.11) | 0.42 | -1.14 (-3.52, 1.24) | 0.35 |
| PSQI (Ref: No sleep disorder) | 0.14 (0.06, 0.22) | < 0.01 | 0.05 (-0.06, 0.16) | 0.35 | 0.62 (-0.78, 2.03) | 0.39 |
| eGFR (mL/min/1.73m²) | 0.00 (0.00, 0.00) | 0.54 | 0.00 (-0.01, 0.00) | 0.35 | -0.02 (-0.07, 0.03) | 0.40 |
| Log BNP | 0.21 (0.1, 0.33) | < 0.01 | 0.00 (-0.16, 0.15) | 0.99 | -0.09 (-2.11, 1.92) | 0.93 |
| IPSS | 0.30 (0.23, 0.37) | < 0.01 | 0.08 (-0.02, 0.18) | 0.13 | 1.23 (-0.07, 2.52) | 0.06 |
| OABSS | 0.30 (0.18, 0.41) | < 0.01 | 0.07 (-0.08, 0.23) | 0.36 | 0.82 (-1.22, 2.86) | 0.43 |
| Nocturnal voiding frequency (times/day) |  |  | -1.87 (-1.93, -1.80) | < 0.01 | -26.27 (-27.16, -25.38) | < 0.01 |
| Log ML exposure | 0.02 (-0.09, 0.13) | 0.76 | 0.17 (0.02, 0.32) | 0.02 | 2.20 (0.26, 4.14) | 0.03 |
| FUSP, the first uninterrupted sleep period; ML, morning light; PSQI, the Pittsburgh Sleep Quality Index; Acti-ODI3%, the actigraphy-modified 3% oxygen desaturation index; eGFR, estimated glomerular filtration rate; BNP, B-type natriuretic peptide; IPSS, the International Prostate Symptom Score; OABSS, the Overactive Bladder Symptom Score. | | | | | | |
