# Supplementary material for: Association between light exposure and sleep problems related to nocturia in older adults: the Nagahama study
Source: J Physiol Anthropol. 2026 Apr 8;45:11. doi: 10.1186/s40101-026-00429-7 (PMC13182062; doi:10.1186/s40101-026-00429-7)
Supplement: Supplementary file 2 — Supplementary Material 2. [file 40101_2026_429_MOESM2_ESM.docx]

| Supplementary table2. Association between sleep problems related to nocturnal urination and the duration of ML exposure ≥ 1,000 lx. | | | | | | |
| --- | --- | --- | --- | --- | --- | --- |
|  | Nocturnal voiding frequency | | FUSP | | FUSP/SPT | |
|  | B (95%CI) | P | B (95%CI) | P | B (95%CI) | P |
| sex (Ref: Male) | -0.12 (-0.22, -0.03) | 0.01 | 0.00 (-0.13, 0.13) | 0.97 | -0.06 (-1.78, 1.67) | 0.95 |
| Age (y) | 0.02 (0.01, 0.03) | < 0.01 | 0.01 (-0.01, 0.02) | 0.39 | 0.08 (-0.08, 0.25) | 0.32 |
| Living arrangement, Living with cohabitants (Ref: Living alone) | 0.03 (-0.13, 0.18) | 0.74 | 0.04 (-0.17, 0.25) | 0.71 | 0.39 (-2.35, 3.14) | 0.78 |
| Educational attainment (y) | -0.01 (-0.03, 0.00) | 0.09 | 0.00 (-0.02, 0.02) | 0.88 | -0.03 (-0.32, 0.26) | 0.85 |
| Household income |  |  |  |  |  |  |
| < 2 million yen | Ref. |  |  |  |  |  |
| 2–4 million yen | 0.00 (-0.09, 0.10) | 0.93 | -0.01 (-0.13, 0.12) | 0.92 | -0.33 (-1.98, 1.31) | 0.69 |
| 4–6 million yen | -0.02 (-0.14, 0.09) | 0.68 | 0.08 (-0.08, 0.24) | 0.35 | 0.33 (-1.77, 2.44) | 0.76 |
| 6–8 million yen | -0.04 (-0.21, 0.12) | 0.58 | 0.10 (-0.12, 0.32) | 0.38 | 0.89 (-1.99, 3.76) | 0.55 |
| ≥ 8 million yen | 0.10 (-0.06, 0.26) | 0.23 | 0.28 (0.06, 0.51) | 0.01 | 2.83 (-0.09, 5.75) | 0.06 |
| Daylight hours (IQR) | 0.00 (-0.03, 0.03) | 0.91 | 0.04 (0.00, 0.09) | 0.05 | 0.6 (0.04, 1.15) | 0.04 |
| Current smoker, Smoking  (Ref: Not Smoking) | -0.17 (-0.33, -0.01) | 0.03 | 0.07 (-0.15, 0.28) | 0.55 | 0.59 (-2.22, 3.39) | 0.68 |
| Drinking frequency (days/week) | 0.00 (-0.02, 0.01) | 0.53 | 0.01 (-0.01, 0.04) | 0.17 | 0.15 (-0.12, 0.42) | 0.27 |
| Physical activity, Regular exercise (Ref: Not regular exercise) | 0.02 (-0.05, 0.09) | 0.59 | -0.01 (-0.11, 0.09) | 0.90 | -0.27 (-1.57, 1.04) | 0.69 |
| BMI | 0.00 (-0.01, 0.01) | 0.82 | 0.00 (-0.01, 0.02) | 0.64 | 0.08 (-0.15, 0.31) | 0.51 |
| Subjective health status,  Good health (Ref: Not health) | 0.08 (-0.02, 0.17) | 0.11 | -0.03 (-0.16, 0.10) | 0.60 | -0.55 (-2.24, 1.15) | 0.53 |
| Diabetes mellitus (Ref: Not diabetes mellitus) | 0.04 (-0.07, 0.15) | 0.44 | -0.08 (-0.23, 0.07) | 0.31 | -1.41 (-3.38, 0.56) | 0.16 |
| Hypertension (Ref: Not hypertension) | 0.03 (-0.04, 0.11) | 0.39 | 0.06 (-0.04, 0.17) | 0.21 | 0.91 (-0.41, 2.23) | 0.18 |
| Sleep medication use (Ref: Not sleep medication use) | -0.02 (-0.14, 0.10) | 0.74 | -0.18 (-0.34, -0.02) | 0.02 | -2.22 (-4.29, -0.15) | 0.04 |
| Sleep onset time (clock time) | -0.24 (-0.28, -0.20) | < 0.01 | -0.54 (-0.6, -0.48) | < 0.01 | 1.60 (0.82, 2.38) | < 0.01 |
| Wake time (clock time) | 0.13 (0.09, 0.18) | < 0.01 | 0.43 (0.37, 0.50) | < 0.01 | -2.93 (-3.76, -2.09) | < 0.01 |
| Log Acti-ODI3% | 0.31 (0.18, 0.45) | < 0.01 | -0.07 (-0.26, 0.11) | 0.43 | -1.13 (-3.51, 1.26) | 0.35 |
| PSQI (Ref: No sleep disorder) | 0.14 (0.06, 0.22) | < 0.01 | 0.05 (-0.06, 0.15) | 0.41 | 0.54 (-0.86, 1.95) | 0.45 |
| eGFR (mL/min/1.73m²) | 0.00 (0.00, 0.00) | 0.54 | 0.00 (-0.01, 0.00) | 0.31 | -0.02 (-0.07, 0.03) | 0.36 |
| Log BNP | 0.21 (0.10, 0.33) | < 0.01 | 0.00 (-0.15, 0.16) | 0.99 | -0.07 (-2.09, 1.95) | 0.95 |
| IPSS | 0.30 (0.23, 0.37) | < 0.01 | 0.08 (-0.02, 0.18) | 0.13 | 1.22 (-0.08, 2.52) | 0.07 |
| OABSS | 0.30 (0.18, 0.41) | < 0.01 | 0.07 (-0.08, 0.23) | 0.36 | 0.82 (-1.22, 2.86) | 0.43 |
| Nocturnal voiding frequency (times/day) |  |  | -1.87 (-1.93, -1.80) | < 0.01 | -26.26 (-27.15, -25.37) | < 0.01 |
| Log the duration of ML exposure ≥ 1,000 lx | 0.02 (-0.09, 0.14) | 0.69 | 0.05 (-0.11, 0.21) | 0.54 | 0.56 (-1.5, 2.62) | 0.59 |
| FUSP, the first uninterrupted sleep period; ML, morning light; PSQI, the Pittsburgh Sleep Quality Index; Acti-ODI3%, the actigraphy-modified 3% oxygen desaturation index; eGFR, estimated glomerular filtration rate; BNP, B-type natriuretic peptide; IPSS, the International Prostate Symptom Score; OABSS, the Overactive Bladder Symptom Score. | | | | | | |
