# Supplementary material for: Association between light exposure and sleep problems related to nocturia in older adults: the Nagahama study
Source: J Physiol Anthropol. 2026 Apr 8;45:11. doi: 10.1186/s40101-026-00429-7 (PMC13182062; doi:10.1186/s40101-026-00429-7)
Supplement: Supplementary file 3 — Supplementary Material 3. [file 40101_2026_429_MOESM3_ESM.docx]

| Supplementary table3. Association between sleep problems related to nocturnal urination and EL exposure. | | | | | | |
| --- | --- | --- | --- | --- | --- | --- |
|  | Nocturnal voiding frequency | | FUSP | | FUSP/SPT | |
|  | B (95%CI) | P | B (95%CI) | P | B (95%CI) | P |
| sex (Ref: Male) | -0.13 (-0.23, -0.04) | 0.01 | -0.01 (-0.14, 0.12) | 0.91 | -0.20 (-1.88, 1.48) | 0.81 |
| Age (y) | 0.02 (0.01, 0.03) | < 0.01 | 0.01 (-0.01, 0.02) | 0.40 | 0.08 (-0.08, 0.24) | 0.34 |
| Living arrangement, Living with cohabitants (Ref: Living alone) | 0.03 (-0.13, 0.18) | 0.72 | 0.04 (-0.17, 0.25) | 0.72 | 0.39 (-2.36, 3.13) | 0.78 |
| Educational attainment (y) | -0.01 (-0.03, 0.00) | 0.09 | 0.00 (-0.02, 0.02) | 0.84 | -0.04 (-0.33, 0.25) | 0.80 |
| Household income |  |  |  |  |  |  |
| < 2 million yen | Ref |  |  |  |  |  |
| 2–4 million yen | 0.01 (-0.09, 0.10) | 0.91 | -0.01 (-0.13, 0.12) | 0.92 | -0.32 (-1.97, 1.32) | 0.70 |
| 4–6 million yen | -0.02 (-0.14, 0.10) | 0.71 | 0.08 (-0.08, 0.24) | 0.35 | 0.34 (-1.76, 2.44) | 0.75 |
| 6–8 million yen | -0.04 (-0.2, 0.12) | 0.61 | 0.10 (-0.12, 0.32) | 0.36 | 0.94 (-1.93, 3.81) | 0.52 |
| ≥ 8 million yen | 0.10 (-0.06, 0.26) | 0.22 | 0.28 (0.06, 0.51) | 0.01 | 2.82 (-0.1, 5.75) | 0.06 |
| Daylight hours (IQR) | 0.00 (-0.03, 0.03) | 0.80 | 0.04 (0.00, 0.09) | 0.05 | 0.61 (0.06, 1.17) | 0.03 |
| Current smoker, Smoking  (Ref: Not Smoking) | -0.17 (-0.33, -0.01) | 0.03 | 0.07 (-0.15, 0.28) | 0.55 | 0.59 (-2.22, 3.39) | 0.68 |
| Drinking frequency (days/week) | 0.00 (-0.02, 0.01) | 0.56 | 0.01 (-0.01, 0.03) | 0.19 | 0.15 (-0.12, 0.42) | 0.29 |
| Physical activity, Regular exercise (Ref: Not regular exercise) | 0.02 (-0.05, 0.09) | 0.54 | 0.00 (-0.1, 0.10) | 0.95 | -0.22 (-1.52, 1.07) | 0.73 |
| BMI | 0.00 (-0.01, 0.01) | 0.79 | 0.00 (-0.01, 0.02) | 0.66 | 0.08 (-0.15, 0.31) | 0.51 |
| Subjective health status,  Good health (Ref: Not health) | 0.08 (-0.02, 0.17) | 0.11 | -0.04 (-0.17, 0.09) | 0.58 | -0.58 (-2.27, 1.12) | 0.50 |
| Diabetes mellitus (Ref: Not diabetes mellitus) | 0.04 (-0.07, 0.15) | 0.44 | -0.08 (-0.23, 0.07) | 0.32 | -1.38 (-3.35, 0.59) | 0.17 |
| Hypertension (Ref: Not hypertension) | 0.03 (-0.04, 0.11) | 0.40 | 0.06 (-0.04, 0.16) | 0.22 | 0.90 (-0.42, 2.22) | 0.18 |
| Sleep medication use (Ref: Not sleep medication use) | -0.02 (-0.13, 0.10) | 0.78 | -0.19 (-0.34, -0.03) | 0.02 | -2.25 (-4.32, -0.18) | 0.03 |
| Sleep onset time (clock time) | -0.24 (-0.28, -0.20) | < 0.01 | -0.55 (-0.61, -0.49) | < 0.01 | 1.58 (0.81, 2.36) | < 0.01 |
| Wake time (clock time) | 0.13 (0.08, 0.17) | < 0.01 | 0.43 (0.37, 0.49) | < 0.01 | -2.98 (-3.8, -2.16) | < 0.01 |
| Log Acti-ODI3% | 0.32 (0.18, 0.45) | < 0.01 | -0.07 (-0.26, 0.11) | 0.42 | -1.13 (-3.51, 1.26) | 0.35 |
| PSQI (Ref: No sleep disorder) | 0.14 (0.06, 0.21) | < 0.01 | 0.04 (-0.06, 0.15) | 0.42 | 0.53 (-0.88, 1.93) | 0.46 |
| eGFR (mL/min/1.73m²) | 0.00 (0.00, 0.00) | 0.56 | 0.00 (-0.01, 0.00) | 0.30 | -0.02 (-0.07, 0.03) | 0.34 |
| Log BNP | 0.22 (0.1, 0.33) | < 0.01 | 0.00 (-0.15, 0.15) | 1.00 | -0.08 (-2.1, 1.94) | 0.94 |
| IPSS | 0.30 (0.23, 0.37) | < 0.01 | 0.08 (-0.02, 0.18) | 0.14 | 1.22 (-0.08, 2.51) | 0.07 |
| OABSS | 0.30 (0.18, 0.41) | < 0.01 | 0.07 (-0.08, 0.23) | 0.35 | 0.84 (-1.20, 2.88) | 0.42 |
| Nocturnal voiding frequency (times/day) |  |  | -1.87 (-1.93, -1.80) | < 0.01 | -26.26 (-27.15, -25.37) | < 0.01 |
| Log EL exposure | -0.06 (-0.18, 0.06) | 0.33 | 0.02 (-0.15, 0.18) | 0.82 | 0.02 (-2.13, 2.18) | 0.98 |
| FUSP, the first uninterrupted sleep period; EL, evening light; PSQI, the Pittsburgh Sleep Quality Index; Acti-ODI3%, the actigraphy-modified 3% oxygen desaturation index; eGFR, estimated glomerular filtration rate; BNP, B-type natriuretic peptide; IPSS, the International Prostate Symptom Score; OABSS, the Overactive Bladder Symptom Score. | | | | | | |
