# Supplementary material for: Association between light exposure and sleep problems related to nocturia in older adults: the Nagahama study
Source: J Physiol Anthropol. 2026 Apr 8;45:11. doi: 10.1186/s40101-026-00429-7 (PMC13182062; doi:10.1186/s40101-026-00429-7)
Supplement: Supplementary file 4 — Supplementary Material 4. [file 40101_2026_429_MOESM4_ESM.docx]

| Supplementary table4. Association between sleep problems related to nocturnal urination and the duration of EL exposure ≥ 50 lx. | | | | | | |
| --- | --- | --- | --- | --- | --- | --- |
|  | Nocturnal voiding frequency | | FUSP | | FUSP/SPT | |
|  | B (95%CI) | P | B (95%CI) | P | B (95%CI) | P |
| sex (Ref: Male) | -0.13 (-0.22, -0.04) | 0.01 | 0.00 (-0.13, 0.13) | 0.99 | -0.14 (-1.82, 1.55) | 0.87 |
| Age (y) | 0.02 (0.01, 0.03) | < 0.01 | 0.01 (-0.01, 0.02) | 0.41 | 0.08 (-0.08, 0.24) | 0.34 |
| Living arrangement, Living with cohabitants (Ref: Living alone) | 0.03 (-0.13, 0.18) | 0.73 | 0.04 (-0.17, 0.25) | 0.73 | 0.39 (-2.36, 3.14) | 0.78 |
| Educational attainment (y) | -0.01 (-0.03, 0.00) | 0.10 | 0.00 (-0.03, 0.02) | 0.80 | -0.04 (-0.33, 0.25) | 0.78 |
| Household income |  |  |  |  |  |  |
| < 2 million yen | Ref |  |  |  |  |  |
| 2–4 million yen | 0.01 (-0.08, 0.10) | 0.87 | -0.01 (-0.14, 0.11) | 0.86 | -0.39 (-2.04, 1.26) | 0.64 |
| 4–6 million yen | -0.02 (-0.14, 0.10) | 0.73 | 0.08 (-0.09, 0.24) | 0.36 | 0.31 (-1.79, 2.42) | 0.77 |
| 6–8 million yen | -0.04 (-0.2, 0.12) | 0.64 | 0.1 (-0.13, 0.32) | 0.39 | 0.83 (-2.05, 3.72) | 0.57 |
| ≥ 8 million yen | 0.10 (-0.06, 0.27) | 0.21 | 0.27 (0.05, 0.50) | 0.02 | 2.72 (-0.20, 5.65) | 0.07 |
| Daylight hours (IQR) | 0.00 (-0.03, 0.03) | 0.88 | 0.04 (0.00, 0.09) | 0.04 | 0.61 (0.06, 1.17) | 0.03 |
| Current smoker, Smoking  (Ref: Not Smoking) | -0.17 (-0.33, -0.01) | 0.03 | 0.07 (-0.15, 0.28) | 0.54 | 0.59 (-2.22, 3.40) | 0.68 |
| Drinking frequency (days/week) | 0.00 (-0.02, 0.01) | 0.59 | 0.01 (-0.01, 0.03) | 0.21 | 0.14 (-0.13, 0.41) | 0.31 |
| Physical activity, Regular exercise (Ref: Not regular exercise) | 0.02 (-0.05, 0.09) | 0.56 | 0.00 (-0.10, 0.10) | 0.94 | -0.23 (-1.53, 1.07) | 0.73 |
| BMI | 0.00 (-0.01, 0.01) | 0.76 | 0.00 (-0.01, 0.02) | 0.72 | 0.07 (-0.16, 0.30) | 0.56 |
| Subjective health status,  Good health (Ref: Not health) | 0.08 (-0.02, 0.17) | 0.12 | -0.03 (-0.16, 0.10) | 0.63 | -0.51 (-2.21, 1.20) | 0.56 |
| Diabetes mellitus (Ref: Not diabetes mellitus) | 0.04 (-0.07, 0.15) | 0.43 | -0.08 (-0.23, 0.07) | 0.32 | -1.39 (-3.36, 0.58) | 0.17 |
| Hypertension (Ref: Not hypertension) | 0.03 (-0.04, 0.10) | 0.41 | 0.07 (-0.04, 0.17) | 0.21 | 0.91 (-0.42, 2.23) | 0.18 |
| Sleep medication use (Ref: Not sleep medication use) | -0.02 (-0.13, 0.10) | 0.79 | -0.19 (-0.35, -0.03) | 0.02 | -2.27 (-4.35, -0.20) | 0.03 |
| Sleep onset time (clock time) | -0.23 (-0.28, -0.19) | < 0.01 | -0.55 (-0.61, -0.49) | < 0.01 | 1.54 (0.75, 2.34) | < 0.01 |
| Wake time (clock time) | 0.13 (0.08, 0.18) | < 0.01 | 0.43 (0.37, 0.49) | < 0.01 | -2.98 (-3.8, -2.16) | < 0.01 |
| Log Acti-ODI3% | 0.31 (0.18, 0.45) | < 0.01 | -0.07 (-0.26, 0.11) | 0.43 | -1.08 (-3.47, 1.31) | 0.38 |
| PSQI (Ref: No sleep disorder) | 0.13 (0.06, 0.21) | < 0.01 | 0.05 (-0.06, 0.16) | 0.38 | 0.57 (-0.83, 1.98) | 0.42 |
| eGFR (mL/min/1.73m²) | 0.00 (0.00, 0.00) | 0.59 | 0.00 (-0.01, 0.00) | 0.32 | -0.02 (-0.07, 0.03) | 0.37 |
| Log BNP | 0.22 (0.10, 0.33) | < 0.01 | 0.00 (-0.16, 0.15) | 0.99 | -0.11 (-2.13, 1.91) | 0.92 |
| IPSS | 0.3 (0.23, 0.37) | < 0.01 | 0.08 (-0.02, 0.18) | 0.13 | 1.21 (-0.09, 2.51) | 0.07 |
| OABSS | 0.29 (0.18, 0.41) | < 0.01 | 0.08 (-0.08, 0.23) | 0.34 | 0.85 (-1.19, 2.89) | 0.41 |
| Nocturnal voiding frequency (times/day) |  |  | -1.86 (-1.93, -1.80) | < 0.01 | -26.25 (-27.14, -25.35) | < 0.01 |
| Log the duration of EL exposure ≥ 50 lx | -0.04 (-0.14, 0.05) | 0.37 | 0.08 (-0.05, 0.21) | 0.22 | 0.60 (-1.09, 2.28) | 0.49 |
| FUSP, the first uninterrupted sleep period; EL, evening light; PSQI, the Pittsburgh Sleep Quality Index; Acti-ODI3%, the actigraphy-modified 3% oxygen desaturation index; eGFR, estimated glomerular filtration rate; BNP, B-type natriuretic peptide; IPSS, the International Prostate Symptom Score; OABSS, the Overactive Bladder Symptom Score. | | | | | | |
