# Supplementary material for: Association between light exposure and sleep problems related to nocturia in older adults: the Nagahama study
Source: J Physiol Anthropol. 2026 Apr 8;45:11. doi: 10.1186/s40101-026-00429-7 (PMC13182062; doi:10.1186/s40101-026-00429-7)
Supplement: Supplementary file 5 — Supplementary Material 5. [file 40101_2026_429_MOESM5_ESM.pptx]

## Slide 1
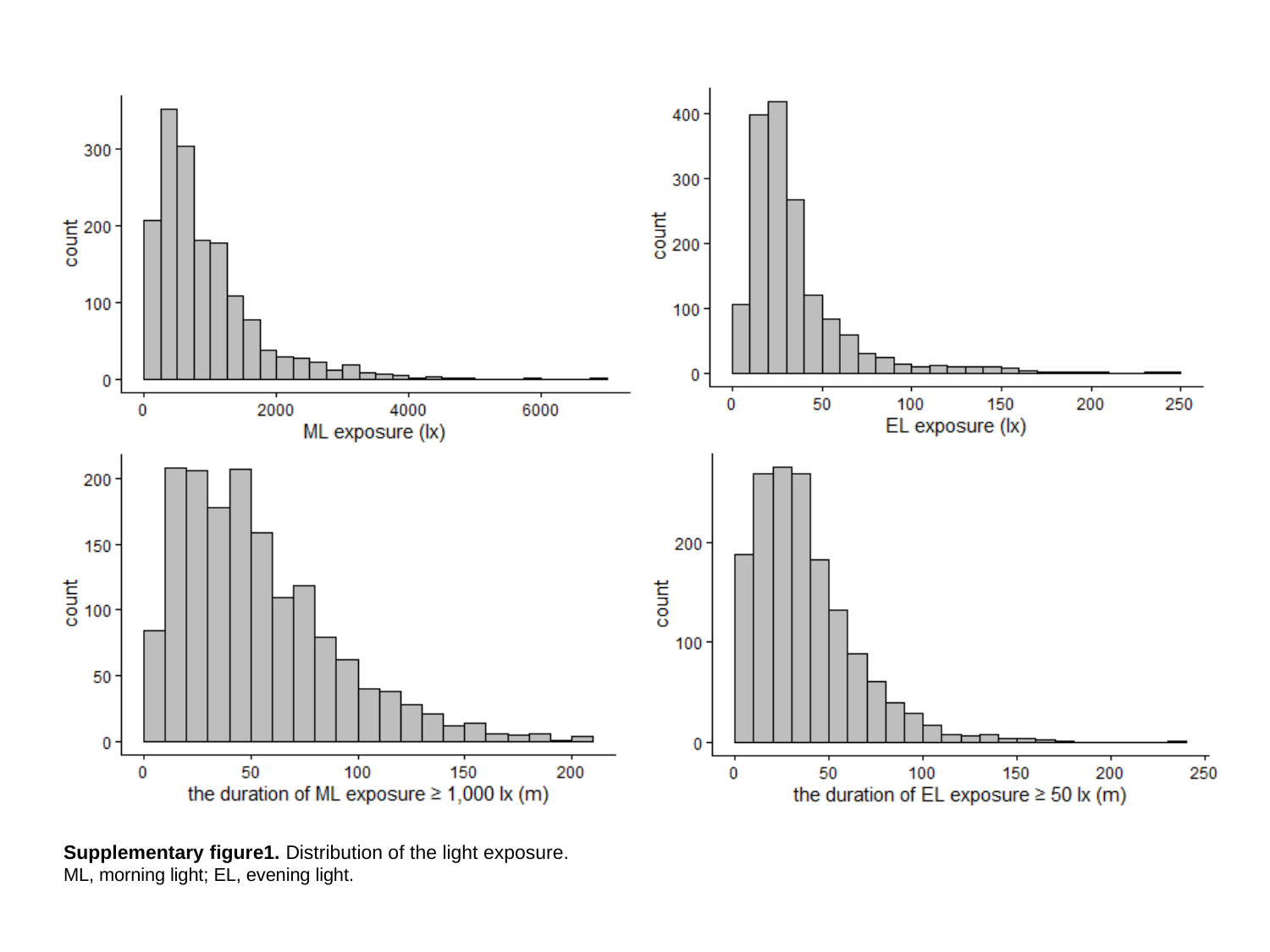

Supplementary figure1. Distribution of the light exposure.
ML, morning light; EL, evening light.
